# Functional characterization and comparative analysis of gene repression-mediating domains interacting with yeast pleiotropic corepressors Sin3, Cyc8 and Tup1

**DOI:** 10.1007/s00294-023-01262-6

**Published:** 2023-03-01

**Authors:** Julia Lettow, Felix Kliewe, Rasha Aref, Hans-Joachim Schüller

**Affiliations:** 1 Center for Functional Genomics of Microbes, Abteilung Molekulare Genetik und Infektionsbiologie, Universität Greifswald, Felix-Hausdorff-Str. 8, 17487 Greifswald, Germany; 2 Present Address: Institut für Anatomie und Zellbiologie, Universitätsmedizin Greifswald, Friedrich-Loeffler-Str. 23c, 17489 Greifswald, Germany; 3 Present Address: Department of Genetics, Faculty of Agriculture, Ain Shams University, Shoubra El-Khaymah, Cairo, 11241 Egypt

**Keywords:** *Saccharomyces cerevisiae*, Gene repression, Corepressor, Sin3, Cyc8, Tup1, Interaction specificity

## Abstract

Transcriptional corepressors Sin3, Cyc8 and Tup1 are important for downregulation of gene expression by recruiting various histone deacetylases once they gain access to defined genomic locations by interaction with pathway-specific repressor proteins. In this work we systematically investigated whether 17 yeast repressor proteins (Cti6, Dal80, Fkh1, Gal80, Mig1, Mot3, Nrg1, Opi1, Rdr1, Rox1, Sko1, Ume6, Ure2, Xbp1, Yhp1, Yox1 and Whi5) representing several unrelated regulatory pathways are able to bind to Sin3, Cyc8 and Tup1. Our results show that paired amphipathic helices 1 and 2 (PAH1 and PAH2) of Sin3 are functionally redundant for some regulatory pathways. WD40 domains of Tup1 proved to be sufficient for interaction with repressor proteins. Using length variants of selected repressors, we mapped corepressor interaction domains (CIDs) in vitro and assayed gene repression in vivo. Systematic comparison of CID minimal sequences allowed us to define several related positional patterns of hydrophobic amino acids some of which could be confirmed as functionally supported by site-directed mutagenesis. Although structural predictions indicated that certain CIDs may be α-helical, most repression domains appear to be randomly structured and must be considered as intrinsically disordered regions (IDR) adopting a defined conformation only by interaction with a corepressor.

**Supplementary Information:**

The online version contains supplementary material available at 10.1007/s00294-023-01262-6.

## Introduction

To prevent gene expression in eukaryotes under adequate conditions, repressor proteins may counteract activators (e. g. by shielding activation domains) or trigger the local formation of a chromatin structure, which is inhibitory against transcriptional activation. Formation of local inactive chromatin can be achieved by recruitment of pleiotropic corepressors associated with histone deacetylases (HDACs; Hildmann et al. 2007). In the yeast *Saccharomyces cerevisiae*, corepressors were genetically identified by isolation of mutations *sin3*, *cyc8* and *tup1* showing complex transcriptional deregulation of several unrelated pathways (reviewed by Grzenda et al. 2009; Malavé and Dent 2006).

Sin3 was initially characterized in yeast as a negative regulator of mating type switch (repressor of *HO*: Swi-independent; Sternberg et al. 1987; Wang et al. 1990) but is strongly conserved in all eukaryotes, being required as an antagonist of cellular proliferation in mammals (Adams et al. 2018). In yeast, several unrelated regulatory systems such as phospholipid biosynthesis, phosphate acquisition, sporulation and silencing of hidden mating type loci are affected by Sin3 (Vidal et al. 1991), leading to a number of alias gene designations. Sin3 is devoid of enzymatic activities but instead functions as a versatile interaction scaffold, using its paired amphipathic helices (PAH1-PAH4; Wang et al. 1990) for binding to pathway-specific repressors (e. g. Ume6 and Opi1; Kadosh and Struhl 1997; Wagner et al. 2001) and HDAC interaction domains (HID; Laherty et al. 1997; Grigat et al. 2012) for recruitment of histone deacetylases. Thus, Sin3 is of central importance for formation of a high-molecular-weight complex of at least 14 subunits (also designated Rpd3L; Carrozza et al. 2005), comprising auxiliary proteins Pho23, Sap30 and Sds3, among others. It has been shown that PAH1 and PAH2 form a bundle of four α-helical segments (also designated “wedged helical bundle”; Spronk et al. 2000), defining a hydrophobic cleft into which Sin3-interaction domains (SID) of repressor proteins can be inserted (Brubaker et al. 2000; Sahu et al. 2008). Importantly, a truncated variant of Sin3A has been identified by exome sequencing of human breast cancer samples, indicating that Sin3A can function as a tumour suppressor in certain tissues (Watanabe et al. 2018).

Cyc8 (= Ssn6) and Tup1 (= Flk1) also negatively influence various regulons in yeast, being required for repression of respiratory functions, glucose-regulated genes, mating functions and DNA damage repair (summarized by Malavé and Dent 2006). Cyc8 and Tup1 form a complex comprised of a Tup1 tetramer, which is associated with a single Cyc8 subunit (Varanasi et al. 1996). Some functional redundancy of Sin3 and Cyc8-Tup1 is supported by the finding of synthetic lethality of mutations *sin3* and *cyc8* (Jäschke et al. 2011). To become recruited to defined target promoters, Cyc8 und Tup1 must interact with specific DNA-binding proteins for which 10 TPR motifs (tetratricopeptide repeat) at the N-terminus of Cyc8 (Schultz et al. 1990; Tzamarias and Struhl 1995) or, presumably, 7 WD40 repeats (= β-transducin repeats; Williams and Trumbly 1990; Komachi et al. 1994) at the C-terminal domain of Tup1 are responsible. Importantly, repression mediated by a LexA-Tup1 fusion is effective even in the absence of Cyc8 while, vice versa, LexA-Cyc8 fails to down-regulate gene expression when Tup1 is missing (Tzamarias and Struhl 1994). It can be concluded that Tup1 is ultimately responsible for gene repression, supported by the finding that Tup1 preferentially binds to underacetylated histones H3 and H4 (Watson et al. 2000). Similar to Sin3, Cyc8 and Tup1 are also able to interact with HDACs (Rpd3, Hda1, Hos1 and Hos2; Wu et al. 2001; Davie et al. 2003). Cyc8-Tup1 may also trigger gene repression by counteracting transcriptional activation (Wong and Struhl 2011). In addition, Tup1 physically interacts with the cyclin-dependent kinase Srb10/Srb11 of the repression-mediating module of the mediator complex (Zaman et al. 2001; Schüller and Lehming 2003). Since *SRB10* and *SRB11* are required for full repression by gene fusions LexA-Cyc8 and LexA-Tup1 (Kuchin and Carlson 1998), Cyc8-Tup1 functionally interferes with the mediator and the RNA polymerase II holoenzyme complex.

Although functional studies of Sin3 and Cyc8-Tup1 mainly focus on gene repression, a simple classification as corepressors would ignore phenotypes which clearly provide evidence also for positive roles. Vidal et al. (1991) comparatively characterized expression of various unrelated genes (such as *PHO5*, *HO*, *STE6* and *SPO11*) in wild-type and *sin3* mutant strains and observed that Sin3 is involved in full repression and maximal activation of these genes, a finding which was also confirmed for genes of phospholipid biosynthesis (Wagner et al. 2001; Kliewe et al. 2017). Similarly, Cyc8-Tup1 can also positively influence the activity of heme-dependent activator Hap1 (Zhang and Guarente 1994), the retrograde-regulated citrate synthase gene *CIT2* (Conlan et al. 1999) and the galactose-inducible *GAL1* gene (Papamichos-Chronakis et al. 2002), once the adequate inducing conditions have become effective. It can be concluded that Sin3, Cyc8 and Tup1 fulfil dual functions as transcriptional corepressors (recruitment of negatively acting HDACs) and as coactivators (recruitment of SAGA; Papamichos-Chronakis et al. 2002; Parnell et al. 2021), depending on the regulatory situation studied.

Since we have previously shown that a pathway-specific repressor such as Opi1 can bind to Sin3 and Cyc8 (Jäschke et al. 2011), we wished to investigate whether other repressors show a similar diversity of interactions. We thus selected various repressor proteins affecting several unrelated regulatory pathways and performed a dual analysis by studying interaction with corepressors in vitro and gene repression in vivo. Our results show that minimal repression domains can be bound by more than a single corepressor while structural predictions provide no precise evidence for a conserved folding pattern common to all repressor proteins tested.

## Materials and methods

### Yeast strains, media and growth conditions

Assays for gene repression in vivo were performed with *S. cerevisiae* strain RTS-lexA, containing an integrated *CYC1-lacZ* reporter gene with 4 lexA operator sites in its upstream region (*MAT*α* leu2 his3 trp1 ura3::lexA*_*Op*_*-CYC1-lacZ::URA3*; Lettow et al. 2022). Transformants with effector plasmids encoding LexA-repressor fusions (based on pRT-lexA: 2 µm *LEU2 MET25*_PR_-HA_3_-lexA_DBD_-NLS) were cultivated under double-selective conditions in synthetic complete media (SCD-Ura-Leu, 2% glucose). Gene repression in vivo was assayed by performing three independent transformations of strain RTS-lexA and using four colonies in each case (12 individual cultivations of transformants and enzyme assays).

For functional studies with Sin3 length variants, isogenic null mutant strain JuLY6 was used (*MAT*α* ura3 leu2 his3 trp1::*ICRE*-CYC1-lacZ::TRP1* ∆*sin3::kanMX*). To assay the regulatory influence of *SIN3* variants, transformants were grown in synthetic complete media with 2% glucose under repressing and derepressing conditions (R: 200 μM inositol + 2 mM choline and D: 5 μM inositol + 5 μM choline), respectively. Specific β-galactosidase activities were measured in 12 independent transformants.

Transformants of the proteinase-deficient strain C13-ABY.S86 (*MAT*α *ura3 leu2 his3 pra1 prb1 prc1 cps1*) were used for preparation of HA-tagged Sin3 length variants.

### Plasmid constructions

GST-fusions of repressor proteins (full-length and truncation variants) were constructed by using the *E. coli* expression plasmid pGEX-2TK (tac promoter-operator; inducible with 1 mM IPTG). For preparation of HA_3_-fusion proteins from *S. cerevisiae* and *E. coli*, expression plasmids p426-MET25HA (promoter inducible by absence of methionine; Mumberg et al. 1994) and pASK-IBA5-HA3 (tet promoter-operator, inducible with 0.2 mg/l anhydrotetracycline; IBA, Göttingen, Germany) were used, respectively.

Using specific primers for yeast repressor genes *DAL80*, *MATα2*,* MIG1*, *MOT3*, *NRG1*, *RDR1*, *ROX1*, *SKO1*, *UME6*, *URE2*, *WHI5*, *XBP1*, *YHP1* and *YOX1*, expression cassettes encoding full-length proteins or length variants were amplified by PCR and used for construction of in-frame fusions with GST (compilation of all plasmids and oligonucleotides in Tables S2 and S3, respectively). These expression cassettes were also used to construct translational lexA-repressor fusions by insertion into the multi-cloning site of plasmid pRT-lexA (episomal plasmid containing *lexA*_DBD_ fused with a nuclear localization sequence and activated by the *MET25* promoter). Plasmids used to study *CTI6*, *FKH1*, *GAL80* and *OPI1* have been described (Aref and Schüller 2020; Aref et al. 2021; Lettow et al. 2022; Jäschke et al. 2011). Epitope-tagged corepressor HA_3_-Sin3 (full-length) was synthesized in yeast or in *E. coli*, using expression plasmids pCW117 (Wagner et al. 2001) or pSW11 (Grigat et al. 2012) while bacterial expression plasmid pJL34 encodes a truncated Sin3 comprising PAH1 and PAH2. Epitope-tagged corepressors Cyc8 and Tup1 were bacterially synthesized using plasmids pFK77 (HA_3_-*CYC8*; encodes aa 1–398 with 10 TPR motifs) and pFK76 (HA_3_-*TUP1*; full-length). Bacterial expression plasmids pVS1, pVS2 and pVS5 encode HA_3_-fusions of Tup1 length variants aa 1–339, aa 340–713 (seven WD40 repeats) and aa 599–713 (WD40 repeats 6 and 7), respectively.

The QuikChange site-directed mutagenesis kit (Agilent) together with pairs of mutagenic primers was used to introduce missense mutations into the *YOX1* coding sequence, replacing selected natural codons against an alanine-specific codon (sequences of mutagenic primers are shown in Supporting Online Table S3). The authenticity of *YOX1* mutational variants was verified by DNA sequencing (LGC Genomics, Berlin, Germany).

### In vitro*-interaction assays*

GST- and HA-modified proteins were synthesized in *E. coli* strain BL21-CodonPlus DE3-RP (Agilent), overproducing selected tRNAs to avoid poor translation.

Crude protein extracts from induced *E. coli* transformants were prepared by sonication. Similar amounts of released GST fusion proteins (according to GST enzyme assays) were bound to glutathione (GSH) sepharose and incubated with yeast or bacterial total protein extracts containing HA fusions of Sin3, Cyc8 or Tup1. Washing conditions and elution of GST fusions together with prey proteins using free GSH have been described (Wagner et al. 2001). Eluted proteins were analyzed by SDS/PAGE and subsequently transferred to a PVDF membrane. HA-tagged proteins could be visualized by treatment with anti-HA-peroxidase conjugate (monoclonal antibody 12CA5 conjugate; Sigma-Aldrich) and POD chemiluminescent substrate, using a digital imager (ChemoStar, Intas).

## Results

### Pathway-specific repressor proteins are able to physically interact with pleiotropic corepressors Sin3, Cyc8 and Tup1

**Fig. 1:**
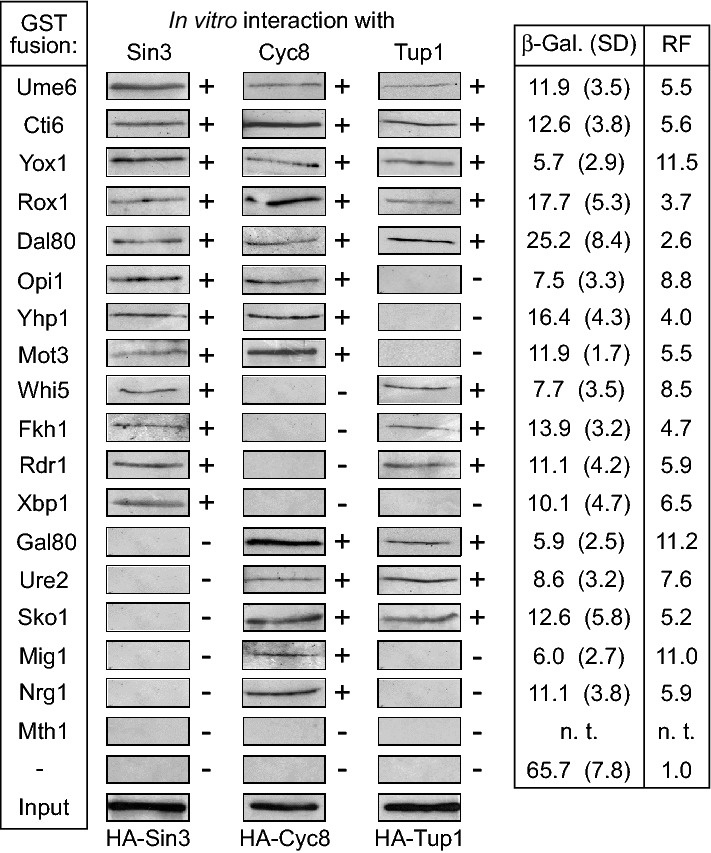
In vitro interaction of selected transcriptional repressor proteins with pleiotropic corepressors and gene repression by lexA fusions in vivo. For pull-down assays, GST fusions with expression cassettes encoding full-length proteins were used (exception: Ume6 aa 508–594, shown to able to bind PAH2 of Sin3; Kadosh and Struhl 1997). GST fusion proteins were immobilized on GSH sepharose and incubated with bacterial protein extracts, containing HA-Sin3 (pSW11; aa 1–1536, full-length), HA-Cyc8 (pFK77, aa 1–398, encoding TPR motifs) or HA-Tup1 (pFK76; full-length). Gene repression in vivo was measured in transformants of strain RTS-lexA (reporter gene lexA_Op_-*CYC1-lacZ*) containing plasmids which encode lexA fusions. Specific β-galactosidase activities are given in nmol oNPG hydrolyzed per min per mg of protein. Protein extracts prepared from at least 12 independent transformants were assayed. GST and lexA fusion plasmids are compiled in Supporting Online Material (Table S2). In vivo gene repression (RF, repression factor) was calculated as the ratio of specific β-galactosidase activities measured in transformants with empty vector pRT-lexA and individual lexA-repressor fusions, respectively. *n. t.* not tested, *SD* standard deviation,  +  in vitro interaction, - no interaction

Transcriptional repressor Opi1 negatively regulates structural genes of yeast phospholipid biosynthesis by interaction with pleiotropic corepressors Sin3 and Cyc8 (Jäschke et al. 2011) both of which are able to counteract gene expression by recruitment of various histone deacetylases. This redundancy of corepressor interaction may be specific for Opi1 but may be also effective for additional repressor proteins. To find out whether functional redundancy is indeed a general mechanism of gene repression, we systematically investigated in vitro interaction of repressors affecting several unrelated regulatory pathways in yeast (Supporting Online Material, Table S1) with corepressors Sin3, Cyc8 and Tup1 by GST pull-down experiments. GST fusions of 18 full-length repressors (exception: Ume6, for which the minimal domain interacting with Sin3 was used) were synthesized in *E. coli*, bound to glutathione sepharose and subsequently incubated with protein extracts containing epitope-tagged corepressors HA-Sin3, HA-Cyc8 and HA-Tup1, respectively. Interaction studies were initially performed with corepressor-containing protein extracts from yeast (not shown) and then repeated with bacterial extracts. These in vitro studies summarized in Fig. 1 confirmed several previously described individual interactions but indeed revealed a complex pattern of interaction between corepressors and repressors some of which may bind three corepressors (5; Ume6, Cti6, Rox1, Yox1 and Dal80), two corepressors (9; Opi1, Yhp1, Mot3, Whi5, Fkh1, Rdr1, Gal80, Ure2 and Sko1) or a single corepressor (3; Xbp1, Mig1 and Nrg1). 13 out of 18 repressor proteins were able to interact with Cyc8, 12 with Sin3 and 11 with Tup1. Eight repressors were able to bind to both subunits of the Cyc8-Tup1 corepressor (Ume6, Cti6, Rox1, Yox1, Dal80, Gal80, Ure2 and Sko1), indicating that redundancy of interaction is effective even within the same complex. These studies were performed with proteins entirely produced by *E. coli*, indicating that direct interactions have been detected in these experiments. Among repressor proteins assayed for interaction, only Mth1 could not bind to a corepressor. Thus, Mth1 was not considered for subsequent investigations. In contrast to a previous publication reporting that Sko1 merely interacts with Tup1 (Pascual-Ahuir et al. 2001) we found that Sko1 could bind Cyc8 and Tup1.

In addition to in vitro binding studies we also investigated whether repressor proteins which can bind at least one corepressor are able to fulfil repressor function in the living cell, thus decreasing gene expression in vivo. An episomal expression plasmid (pRT-lexA) containing a *MET25*_Prom_-HA-*lexA*_DBD_-NLS cassette with a versatile multi-cloning site was constructed and used to generate lexA_DBD_-repressor fusions. To assay gene repression in vivo, derivatives of pRT-lexA encoding lexA_DBD_-repressor fusions were transformed into a yeast strain containing an integrated reporter gene with a lexA operator sequence upstream of UAS1 and UAS2 of a *CYC1-lacZ* promoter fusion (Kadosh and Struhl 1997). Once recruited to the lexA_Op_ upstream of the native *CYC1* promoter, a functional repressor should significantly decrease expression of the reporter gene *lacZ*, leading to reduced activity of β-galactosidase. As is also shown in Fig. 1, lexA_DBD_-repressor fusions were indeed able to downregulate expression of the reporter gene, although to varying degrees (2.6-fold repression mediated by Dal80 vs. 11.5-fold repression by Yox1). As is evident from the comparison of Yox1 and Dal80, which may both bind to Sin3, Cyc8 and Tup1, the strength of gene repression in vivo does not simply correlate with the number of corepressors a given repressor may contact.

### Sin3-binding repressor proteins interact with PAH1 or PAH2

Although the four PAH domains of Sin3 were proposed as sites of protein–protein-interactions (Wang et al. 1990), previous studies with a limited number of proteins indicated that PAH1 and PAH2 are mainly responsible for repressor recruitment (Wagner et al. 2001; Washburn and Esposito 2001; Sahu et al. 2008). We thus systematically investigated which of its domains is contacted by 10 repressor proteins able to bind full-length Sin3 in vitro (not shown: Ume6 for which PAH2 was described as its target site, Washburn and Esposito 2001; Cti6, which binds to PAH1 and PAH2, Aref and Schüller 2020).

**Fig. 2:**
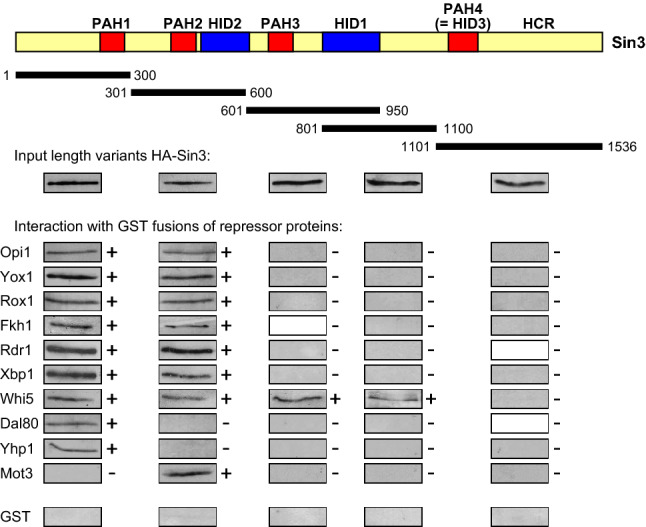
Interaction of gene-specific repressor proteins with functional domains of Sin3. GST fusions with full-length repressor proteins (for expression plasmids cf. Supporting Online Table S2) were immobilized on GSH sepharose and incubated with protein extracts from yeast transformants, containing epitope-tagged Sin3 length variants 1–300 (PAH1; pCW83), 301–600 (PAH2; pYJ91), 601–950 (PAH3 + HID1; pYJ90), 801–1100 (HID1; pYJ89) and 1100–1536 (PAH4; pMP20), respectively. GST devoid of repressor fusion served as a negative control. *HID* HDAC interaction domain; *PAH*, paired amphipathic helix,  +  in vitro interaction, - no interaction

Epitope-tagged length variants of Sin3 were synthesized in *S. cerevisiae* and protein extracts subsequently used for interaction studies with immobilized GST fusions of repressor proteins. As is depicted in Fig. 2, several repressors are able to bind PAH1 and PAH2 in vitro (Opi1, Yox1, Rox1, Fkh1, Rdr1, Xbp1 and Whi5) while others show specificity for PAH1 (Dal80, Yhp1) or PAH2 (Mot3). As an exception, Whi5 could also interact with additional Sin3 length variants both of which contain the HDAC interaction domain 1, known to recruit the yeast major HDAC Rpd3 (Laherty et al. 1997; Grigat et al. 2012).

Although we have previously found that Opi1 contacts PAH1 of Sin3 (Wagner et al. 2001), these results show that PAH2 can be bound as well. To obtain evidence for the in vivo significance of Opi1-Sin3 interactions, we investigated regulated expression of a reporter gene dependent on the Opi1-controlled UAS element of phospholipid biosynthetic genes (ICRE-*CYC1-lacZ*, inositol/choline response element; Wagner et al. 2001) in the presence of several Sin3 deletion variants (Wang and Stillman 1993). As is shown in Table 1, our assays confirmed the severe deregulation of ICRE-dependent gene expression in a *sin3* deletion strain (increased expression under repressing conditions, decreased expression under derepressing conditions). Removal of each single PAH from Sin3 still allowed a significant repression of the reporter gene while gene expression in the absence of both PAH1 and PAH2 was substantially increased. It should be mentioned that loss of PAH1 also prevents full derepression of the ICRE-dependent reporter gene. In summary, we conclude that PAH1 and PAH2 are indeed redundant for mediating Opi1-dependent gene repression. A similar functional redundancy may be also effective for other repressors shown to bind in vitro to both domains, PAH1 and PAH2.

**Table 1:** Influence of Sin3 deletion variants on regulated expression of an ICRE-dependent reporter gene

Plasmid genotype	Specific β-gal. activity		D/R
	R (SD)	D (SD)	
–	28.4 (4.4)	33.7 (7.3)	1.2
*SIN3* (wild-type)	4.9 (0.8)	49.2 (11.9)	10.0
∆PAH1	4.9 (1.5)	28.9 (8.7)	5.9
∆PAH2	5.5 (2.7)	51.9 (18.7)	9.4
∆(PAH1 + PAH2)	19.0 (5.8)	30.9 (10.3)	1.6
∆PAH3	5.8 (2.7)	44.8 (11.2)	8.8
∆PAH4	5.7 (2.1)	47.2 (13.4)	8.3
∆(PAH4 + HCR)	8.9 (3.6)	52.5 (13.7)	5.4

*SIN3* encoding single-copy plasmids (*ARS CEN URA3*) were transformed into strain JuLY6 (*ura3 sin3*∆ ICRE*-CYC1-lacZ*). Transformants were grown under repressing (R, 200 μM inositol + 2 mM choline) and derepressing conditions (D, 5 μM inositol + 5 μM choline). Specific β-galactosidase activities are given in nmol oNPG hydrolyzed per min per mg of protein

*HCR* highly conserved region, *PAH* paired amphipathic helices, *SD* standard deviation

### Tup1-binding repressor proteins interact with WD40 repeats

**Fig. 3:**
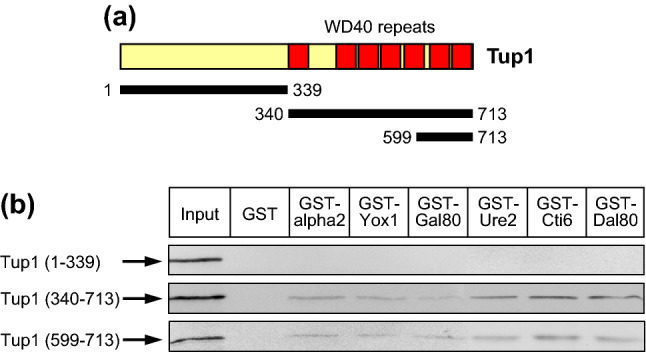
Interaction of selected gene-specific repressor proteins with N- and C-terminal length variants of Tup1. **a** Structural features of Tup1 and position of HA-tagged length variants used for interaction studies. **b** GST fusions with repressor proteins alpha2, Yox1, Gal80, Ure2, Cti6 and Dal80 (for expression plasmids cf. Supporting Online Table S2) were immobilized on GSH sepharose and incubated with bacterial protein extracts, containing epitope-tagged Tup1 length variants 1–339 (no WD40 repeats; pVS1), 340-713 (WD40 repeats 1–7; pVS2) and 599–713 (WD40 repeats 6 and 7; pVS5), respectively

It has been shown that repressor interactions with Cyc8 are mediated by TPR domains clustered at its N-terminus (Schultz et al. 1990; Tzamarias and Struhl 1994, 1995; Jäschke et al. 2011). Although WD40 repeats of Tup1 are generally presumed to mediate interaction with repressor proteins, experimental evidence for this assumption has been reported only in details for alpha2 (α2; Komachi et al. 1994). To investigate whether Tup1 recruitment by other repressors is exclusively dependent on its WD40 domain, we generated Tup1 length variants separating the N-terminus responsible for binding of Cyc8, mediator and histones H3/H4 (aa 1–339) from the C-terminus containing seven WD40 repeats (aa 340–713). We also constructed a Tup1 truncation encoding only two complete WD40 repeats (aa 599–713; WD40-6 and -7; Fig. 3a). Using alpha2 as a reference, we could indeed show that Yox1, Gal80, Ure2, Cti6 and Dal80 are able to bind to WD40 repeats but not to the N-terminus of Tup1 (Fig. 3b). Repressor-Tup1 interaction does not require the entire set of seven WD40 repeats but is also effective with only two WD40 repeats, confirming a previous study which showed that alpha2 can bind to WD40-2 (Komachi et al. 1994). We conclude that WD40 repeats exhibit only a limited degree of specificity but are in fact substantially redundant for repressor interaction.

### Mapping and mutational analysis of corepressor binding domains

We could previously show that a single domain within repressor Opi1 mediates contact to both corepressors Sin3 and Cyc8 (OSID, aa 1–106) and that Opi1 OSID missense variants identically affect interaction with Sin3 and Cyc8 (Jäschke et al. 2011). To identify minimal domains within repressors involved in repressor-corepressor interaction and possibly to derive common sequence patterns we constructed GST fusions encoding length variants of repressors Yox1, Dal80, Xbp1, Ure2, Rox1, Mot3, Rdr1 and Sko1, which were used for binding studies with epitope-tagged corepressors. Similar studies have been previously described for Mig1 (Östling et al. 1996), Ume6 (Kadosh and Struhl 1997), Cti6 (Aref and Schüller 2020), Fkh1 (Aref et al. 2021) and Gal80 (Lettow et al. 2022). No mapping studies were performed with Nrg1, Yhp1 and Whi5.

#### Yox1

**Fig. 4:**
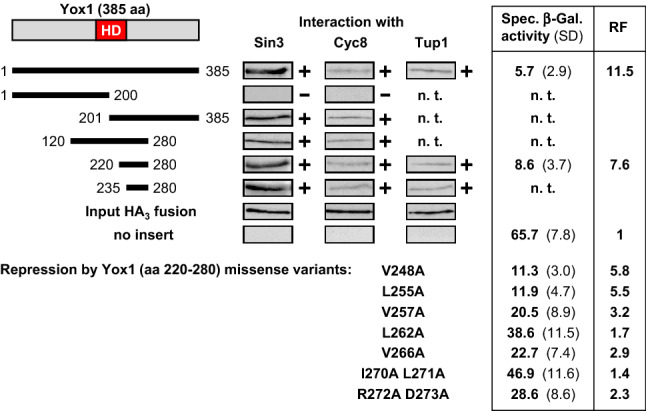
Molecular analysis of Yox1 interaction with corepressors and gene repression by lexA fusions. Yox1 length variants were fused with GST, immobilized on GSH sepharose and incubated with bacterial protein extracts, containing HA-Sin3 (pJL34; aa 1-480, comprising PAH1 and PAH2), HA-Cyc8 (pFK77, aa 1-398, encoding TPR motifs) or HA-Tup1 (pFK76; full-length). GST expression plasmids and lexA fusion plasmids encoding length variants or missense mutations of Yox1 are compiled in Supporting Online Material (Table S2). Gene repression in vivo was measured in transformants of strain RTS-lexA (reporter gene lexA_Op_-*CYC1-lacZ*). Empty vector pRT-lexA devoid of effector domains was used as a negative control for maximal reporter gene expression. Specific β-galactosidase activities are given in nmol oNPG hydrolyzed per min per mg of protein. Protein extracts prepared from at least 12 independent transformants were assayed. *HD* homeodomain, *n. t.* not tested, *RF* repression factor, *SD* standard deviation,  +  in vitro interaction, - no interaction

The homeodomain-containing repressor Yox1 is phosphorylated by the cyclin-dependent kinase Cdc28/Cdk1 and counteracts gene activation by the MADS box transcription factor Mcm1, which is responsible for expression of genes with an ECB promoter motif (early cell cycle box; Pramila et al. 2002). Since Yox1 mediated strong gene repression in vivo and interacted with corepressors Sin3, Cyc8 and Tup1 (Fig. 1), we performed a detailed analysis of its molecular functions. Length variants of Yox1 were fused with GST and incubated with bacterial protein extracts containing Sin3, Cyc8 and Tup1, respectively. As is shown in Fig. 4, a short region comprising aa 220–280 of Yox1 following its homeodomain was able to interact with Sin3, Cyc8 and Tup1 in vitro and could also repress expression of a lexA_Op_-dependent reporter gene in vivo when fused with LexA. Corepressor interaction was also observed with an even shorter Yox1 domain (aa 235–280). For a more precise analysis, we introduced missense mutations into Yox1 length variant aa 220–280 at selected positions, focusing on several hydrophobic amino acids at positions reminiscent of a heptad-like sequence pattern. Gene repression was strongly weakened when residues V257, L262, V266 and I270 L271 were replaced with alanine (Fig. 4). As is evident from a double mutation replacing R272 D273, not only hydrophobic residues are important for functional repression, indicating that at least Yox1 aa 257–273 define the functional core of its repression domain.

#### Dal80

The zinc finger repressor Dal80 (degradation of allantoin; Cunningham and Cooper 1993) negatively regulates genes required for acquisition of nitrogen from poor sources by binding to URS_GATA_ sequence motifs but its mechanism of repression has not been described previously. As demonstrated above (Fig. 1), Dal80 can interact with corepressors Sin3, Cyc8 and Tup1.

**Fig. 5:**
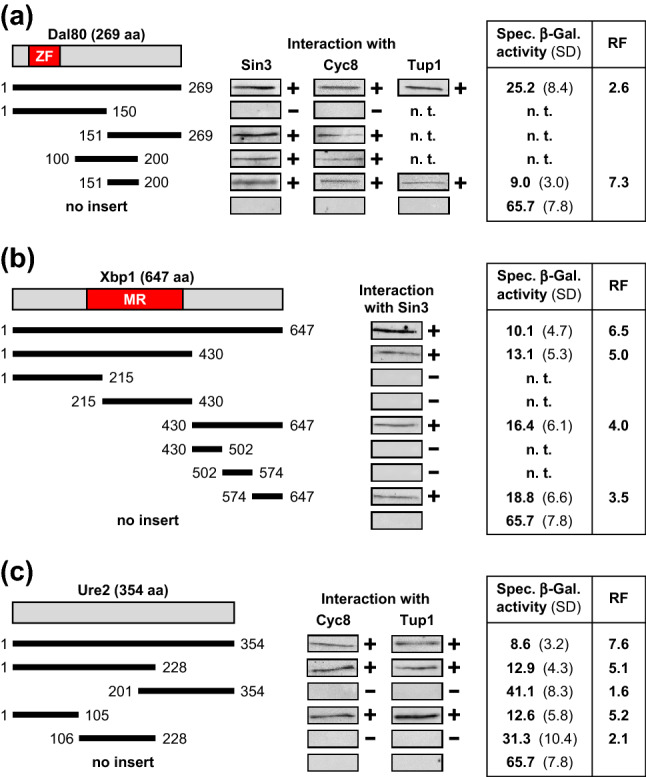
Molecular analysis of corepressor interaction in vitro and gene repression in vivo by lexA fusions of repressors Dal80 (**a**), Xbp1 (**b**) and Ure2 (**c**). Length variants of repressor proteins were fused with GST, immobilized on GSH sepharose and incubated with bacterial protein extracts, containing HA-Sin3 (pJL34; aa 1-480, comprising PAH1 and PAH2), HA-Cyc8 (pFK77, aa 1-398, encoding TPR motifs) or HA-Tup1 (pFK76; full-length). GST expression plasmids and lexA fusion plasmids encoding length variants of Dal80, Xbp1 and Ure2 are compiled in Supporting Online Material (Table S2). Gene repression in vivo was measured in transformants of strain RTS-lexA (reporter gene lexA_Op_-*CYC1-lacZ*) which contain plasmids encoding lexA fusions. Empty vector pRT-lexA was used as a negative control for maximal reporter gene expression. Specific β-galactosidase activities are given in nmol oNPG hydrolyzed per min per mg of protein. Protein extracts prepared from at least 12 independent transformants were assayed. *ZF* zinc finger, *MR* Mbp1-related DNA-binding domain, *n. t.* not tested, *RF* repression factor, *SD* standard deviation,  +  in vitro interaction, - no interaction

GST fusions of Dal80 length variants showed that its N-terminal part comprising the zinc finger domain was unable to interact with corepressors (Fig. 5a). In contrast, truncations representing the C-terminus and internal sequences could bind to Sin3 and Cyc8. Indeed, aa 151–200, which are common to these truncations were able to interact with three corepressors and could also mediate strong gene repression in vivo (repression factor 7.3), which is substantially more effective than observed with entire Dal80. Possibly, full-length Dal80 contains sequences counteracting maximal gene repression executed by the minimal core domain aa 151–200.

#### Xbp1

The DNA-binding repressor Xbp1 prevents expression of cyclin genes of the G1 phase and thus causes delay of cell cycle progression (Mai and Breeden 2000). Xbp1 contains an Mbp1-related DNA-binding domain and was shown to interact with Sin3 but not with Cyc8 and Tup1 (Fig. 1). In contrast to other repressor proteins studied here, Xbp1 contains at least two non-overlapping domains mediating corepressor interaction and gene repression in vivo (aa 1–430 and aa 430–647; Fig. 5b). Using additional Xbp1 truncations, we could finally map a repression domain of 74 aa at the ultimate C-terminus of the protein. No further mapping was done for the length variant aa 1–430.

#### Ure2

This negative regulator of nitrogen catabolite repression is devoid of an obvious DNA-binding domain but contains two domains related to glutathione-S-transferases (GST) of unknown significance. In the presence of a favorable nitrogen source, Ure2 prevents gene activation by GATA factors Gln3 and Gat1 (Cooper 2002). Ure2 can convert into the conformational variant [URE3], forming polymeric amyloid prion-like filaments being able to self-propagate but unable to mediate gene repression (Baxa et al. 2002). Interestingly, the prion-forming domain of Ure2 (aa 1–80; Baxa et al. 2002) is part of its Cyc8 and Tup1 interaction domain, which also mediates efficient gene repression in vivo (5.2-fold; Fig. 5c). In contrast, GST-related domains of Ure2 (aa 106–228 and aa 201–354) were unable to bind corepressors.

#### Rox1

Oxygen limitation (hypoxia) requires activation of certain respiratory enzymes such as cytochrome and cytochrome oxidase which, however, are repressed by Rox1 under aerobic conditions. Rox1 contains an N-terminal HMG box sequence motif (high mobility group) mediating its binding to promoter sites upstream of hypoxic genes such as *ANB1* and *CYC7* (Deckert et al. 1999). Rox1 is able to interact with corepressors Sin3, Cyc8 and Tup1 in vitro, utilizing a repression domain comprising amino acids 124–200 (Supporting Online Fig. 1a).

#### Mot3

Similar to Rox1, the zinc finger protein Mot3 is required for repression of certain hypoxic genes among which are genes of ergosterol biosynthesis (Hongay et al. 2002). As shown above (Fig. 1), Mot3 binds to Cyc8 and Tup1 in vitro, mediated by two non-overlapping domains before and behind its zinc finger motif. However, efficient repression in vivo can be observed only with aa 231–347, which are thus considered as a genuine repression domain (Supporting Online Fig. 1b).

#### Rdr1

*PDR5* encoding an ABC transporter of the plasma membrane responsible for efflux of various drugs is negatively regulated by the Zinc cluster repressor Rdr1 (Hellauer et al. 2002). Our mapping results show that an internal domain comprising aa 364–455 of Rdr1 mediate interaction with Sin3 (Supporting Online Fig. 1c).

#### Sko1

Initially, Sko1 was identified as a negative regulator of the invertase gene *SUC2* with a CREB-related leucine zipper (Nehlin et al. 1992). However, its major function is regulation of genes required to counteract hyperosmotic stress (such as the sodium exporter *ENA1*). Importantly, Sko1 can be converted into an activator by Hog1-dependent phosphorylation, which is now able to recruit complexes SAGA and SWI/SNF to its target promoters (Proft and Struhl 2002). In this work we demonstrate that Sko1 interaction with Cyc8 requires a central domain (aa 201–427), which also mediates gene repression in vivo (Supporting Online Fig. 1d).

Using experimental results from this work and mapping data previously described, we now can compile sequences responsible for corepressor recruitment from 16 repressor proteins of *S. cerevisiae* (Table 2).

**Table 2:** Compilation of corepressor-recruiting and gene repression-mediating domains within 16 transcriptional repressor proteins of *S. cerevisiae*

Repressor	Position	Sequence
alpha2	1–70	MNKIPIKDLLNPQITDEFKSSILDINKKLFSICCNLPKLPESVTTEEEVE LRDILGFLSRANKNRKISDE
Cti6	450–506	QSDREEFVRFVENQHFVEKVDTIYNGYNESLSMMDDLTRELLLWEKKYSN NTNAIQ
Dal80	151–200	ECSTQRGKFSLDPCEPSGKNYLYQINGSDIYTSNIELTRLPNLSTLLEPS
Fkh1	51–160	SIAREVNAYAKIAGCDWTYYVQKLEVTIGRNTDSLNLNAVPGTVVKKNID IDLGPAKIVSRKHAAIRFNLESGSWELQIFGRNGAKVNFRRIPTGPDSPP TVLQSGCIID
Gal80	81–145	FASSSTIDMIVIAIQVASHYEVVMPLLEFSKNNPNLKYLFVEWALACSLD QAESIYKAAAERGVQ
Mig1	481–504	DSQVQELETLPPIRSLPLPFPHMD
Mot3	231–347	APAPAPGPPHHHHHHSNTHNNLNNGGAVNTNNAPQHHPTIITDQFQFQLQ QNPSPNLNLNINPAQPLHLPPGWKINTMPQPRPTTAPNHPPAPVPSSNPV ASNLVPAPSSDHKYIHQ
Opi1	1–106	MSENQRLGLSEEEVEAAEVLGVLKQSCRQKSQPSEDVSQADKMPASESST TPLNILDRVSNKIISNVVTFYDEINTNKRPLKSIGRLLDDDDDEHDDYDY NDDEFF
Rdr1	364–455	PQDSVNANAAQLLQALAAVHESPNAHPFLSLTKGDICLSLYRRLRLLNHI LDKNVVLQIIDIGNTALSAAYALVKLDQAWWNVLSTSFQYVC
Rfx1 (= Crt1)	1–130	MVIFKERKPTENLFTRKIPAKYFIFSPSFLSVHYFEFYLPMSGDNNIEPT SRGSNDNSNGPSNGSSVNSNRYSLNAPKYSSQPPPASHTYLPPMSVNIPP IASKSSSIYSLLHQSSPRPETPNPILPPLI
Rox1	124–200	PFNNNIVLMKRAHSLSPSSSVSSSNSYQFQLNNDLKRLPIPSVNTSNYMV SRSLSGLPLTHDKTARDLPQLSSQLNS
Sko1	270–427	IMHPTVNGTPLTPGLSSLLNLPSTGVLANPVFKSTPTTNTTDGTVNNSIS NSNFSPNTSTKAAVKMDNPAEFNAIEHSAHNHKENENLTTQIENNDQFNN KTRKRKRRMSSTSSTSKASRKNSISRKNSAVTTAPAQKDDVENNKISNNV TLDENEE
Ume6	508–594	SASSSTKLDDDLGTAAAVLSNMRSSPYRTHDKPISNVNDMNNTNALGVPA SRPHSSSFPSKGVLRPILLRIHNSEQQPIFESNNST
Ure2	1–105	MMNNNGNQVSNLSNALRQVNIGNRNSNTTTDQSNINFEFSTGVNNNNNNN SSSNNNNVQNNNSGRNGSQNNDNENNIKNTLEQHRQQQQAFSDMSHVEYS RITKF
Xbp1	574–647	RHYNVPSSPIAPAPPTFPQPYGDDHYHFLKYASEVYKQQNQRPAHNTNTN MDTSFSPRANNSLNNFKFKTNSKQ
Yox1	235–280	RIATSKSTTIIQTVSPPSPPLDVHATPLASRVKADILRDGSSCSRSSSSS PLENTP

Amino acid residues reminiscent of a heptad-like hydrophobic-amphipathic sequence pattern are underlined. Data for alpha2, Cti6, Fkh1, Gal80, Mig1, Opi1, Rfx1 and Ume6 were taken from Komachi et al. (1994), Aref and Schüller (2020), Aref et al. (2021), Lettow et al. (2022), Östling et al. (1996), Jäschke et al. (2011), Zhang and Reese (2005) and Kadosh and Struhl (1997), respectively

## Discussion

In this work we investigated 18 selected pathway-specific transcriptional repressor proteins for their diversity of interactions with pleiotropic corepressors Sin3, Cyc8 and Tup1. As previously shown for the intensively characterized negative regulator Opi1 of phospholipid biosynthesis (Jäschke et al. 2011), most repressors are able to contact more than a single corepressor. Principally, such versatile repressors could (I) use several distinct interaction domains or (II) use the same domain for all of these interactions. Previously, a separation of functions has been described for alpha2 being responsible for repression of a- and haploid-specific genes in *S. cerevisiae*. While the N-terminal repression-mediating domain of alpha2 interacts with various WD40 repeats of Tup1 (Komachi et al. 1994), TPR motifs of Cyc8 can bind to the C-terminal homeo domain of alpha2 (Smith et al. 1995). However, results of mapping studies reported here and in previous work (Aref and Schüller 2020; Lettow et al. 2022) provide clear evidence for the idea that functional minimal domains some of which are no longer than 50–60 aa as shown for Yox1 and Dal80 can interact with Sin3, Cyc8 and Tup1. Such corepressor interaction domains (CID) must be able to contact α-helical structures of PAH motifs in Sin3 (four-helix bundle; Brubaker et al. 2000; Sahu et al. 2008), TPR motifs of Cyc8 forming two α -helical segments (helix-turn-helix; D'Andrea and Regan 2003) as well as WD40 motifs of Tup1 exhibiting a β-sheet of a seven-bladed propeller structure (Sprague et al. 2000). Consequently, a considerable structural flexibility and malleability of CIDs within repressor proteins is required. Indeed, circular dichroism (CD) experiments showed that CID peptides are unfolded in the absence of corepressors while a defined secondary structure is adopted in their presence (forming amphipathic α -helices together with Sin3; Spronk et al. 2000; Swanson et al. 2004; He et al. 2021). It can be concluded that CIDs are intrinsically disordered regions (IDRs) lacking a precise three-dimensional structure which, however, perform a disorder-to-order transition once their binding partners are present (Cumberworth et al. 2013).

Results of mapping studies described here and elsewhere allowed us to compile 16 sequences of corepressor interaction domains being able to contact Sin3, Cyc8 and/or Tup1 for mediating pathway-specific gene repression (Table 2). We thus wished to comparatively analyze and possibly to derive sequence motifs, which are common to CIDs. Focusing on Sin3 interaction domains (SID), a limited number of repressor-corepressor interactions were previously investigated structurally. These NMR studies led to several related consensus motifs, emphasizing the importance of hydrophobic residues with a defined spatial pattern (Φ = bulky hydrophobic residue; X = any nonproline residue; A = Ala; L = Leu):

SID of Mad1 binding to PAH2 of mSin3A: ΦXXΦXAAXXΦ (Brubaker et al. 2000);

SID of HBP1 binding to PAH2 of mSin3A: AAX ΦXXΦ (Swanson et al. 2004);

SID of REST binding to PAH1 of mSin3B: LXXL ΦXΦA (Nomura et al. 2005);

SID of Sap25 binding to PAH1 of mSin3A: type I, ΦXΦXXΦ; type II, ΦXXΦXΦ (Sahu et al. 2008);

SID of Pf1 binding to PAH2 of mSin3A: ΦXXΦXAA (Kumar et al. 2011);

SID of Myt1L binding to PAH1 of mSin3B: ΦXXΦXΦ (Marcum and Radhakrishnan 2020);

SID of TGIF1 binding to PAH2 of mSin3A: ΦXXLΦXΦA; (He et al. 2021).

Using the data of Table 2, we thus searched for sequence patterns exhibiting hydrophobic residues at various defined positions of a putative heptad motif (1-4-7; 1-4-1; 1-5-7; 1-5-1 and 1-5-2; for details see compilation in Table 3). At least a single sequence pattern could be identified within each of the 16 CIDs compiled while some of them match with all patterns (alpha2, Fkh1, Opi1, Rdr1 and Rfx1). Mutational analysis of hydrophobic amino acids within the single 1-5-2 pattern of Yox1 indeed confirmed their importance for gene repression in vivo (Fig. 4). However, not only hydrophobic but also charged residues such as Arg or Asp contribute to functional repression by Yox1. Electrostatic interactions have been also described for Mad1-mSin3B (salt bridge between Mad1 E23 and PAH2 K165; van Ingen et al. 2004) and TGIF1-mSin3A (salt bridge between TGIF1 E394 and PAH2 K326; He et al. 2021). Polar interactions by hydrogen bonding were identified for complexes TGIF1-mSin3A (TGIF1 Q380 and PAH2 Q336; He et al. 2021) and Pf1-mSin3A (Pf1 N221 and PAH2 K315/Y325).

**Table 3:**
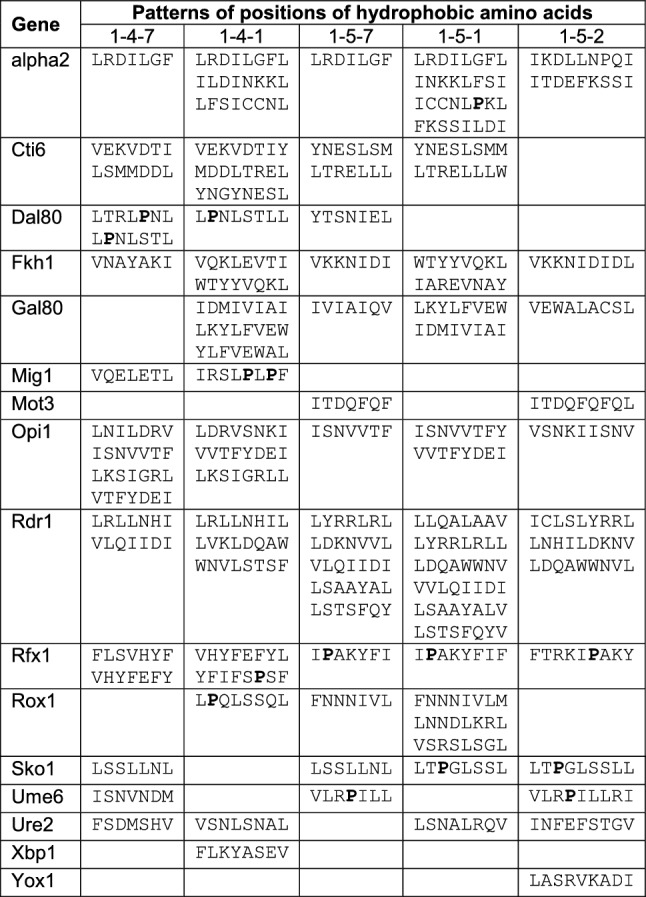
Compilation of sequence motifs from corepressor interaction domains exhibiting periodic heptad patterns of hydrophobic amino acids (data derived from Table 2)

As an example, 1–4-1 means that hydrophobic residues are found at positions 1 and 4 of the first heptad and position 1 of the next heptad. Some sequence motifs partially overlap or agree with more than a single pattern and are thus shown repeatedly. Proline residues are depicted in bold

Although the anti-parallel orientation of the two α-helices forming a single TPR motif partially resembles the wedged helix bundle typical for PAH1 and PAH2, much less is known about specific sequence requirements of TPR ligand binding (reviewed by Zeytuni and Zarivach 2012). Both domains form an independent folding unit with a conformation which is not substantially affected by binding of their CID ligands (Brubaker et al. 2000; D'Andrea and Regan 2003). The complex of a model peptide (MEEVD, representing the C-terminus of Hsp90) with an artificially designed TPR module was studied by X-ray crystallography, providing evidence for a combined influence of hydrophobic (M, V) and electrostatic interactions (E, D). Acidic residues are bound by a “carboxylate clamp”, established by residues K, N and R within the TPR module (Cortajarena et al. 2010).

WD40 motifs with a seven-bladed propeller structure and blades formed by four anti-parallel β-sheets may exhibit the largest degree of structural versatility for ligand binding. Data summarized by Stirnimann et al. (2010) and Xu and Min (2011) show that distinct regions of the propeller can be involved in binding of partner proteins which may contain α-helical segments but can be also unstructured.

To compare conformations of repressors investigated here and elsewhere we finally used structure predictions of *S. cerevisiae* proteins by the recently described bioinformatic tool Alphafold (Jumper et al. 2021), focusing on their respective CIDs. As is shown in Supporting Online Fig. 2, no uniform structural motifs are detectable. While α-helices are dominant only within CIDs of Cti6, Opi1 and Rdr1, random structures partially mixed with helical or sheet motifs characterize CIDs of the remaining repressor proteins. As mentioned above, it appears plausible that most corepressor interaction domains are intrinsically disordered and become locally ordered only in the presence of a corepressor template providing a defined structural framework (“grafting”).

## Supplementary Information

Below is the link to the electronic supplementary material.

Supplementary file1 (DOCX 1912 KB)

## Data Availability

Original data are available upon request. Additional information is provided in the Supplementary Material.
