# Supplementary material for: Functional characterization and comparative analysis of gene repression-mediating domains interacting with yeast pleiotropic corepressors Sin3, Cyc8 and Tup1
Source: Curr Genet. 2023 Mar 1;69(2-3):127–39. doi: 10.1007/s00294-023-01262-6 (PMC10163088; doi:10.1007/s00294-023-01262-6)
Supplement: Supplementary file 1 — Supplementary file1 (DOCX 1912 KB) [file 294_2023_1262_MOESM1_ESM.docx]

**Table 3** Compilation of sequence motifs from corepressor interaction domains exhibiting periodic heptad patterns of hydrophobic amino acids (data derived from Table 2). As an example, 1-4-1 means that hydrophobic residues are found at positions 1 and 4 of the first heptad and position 1 of the next heptad. Some sequence motifs partially overlap or agree with more than a single pattern and are thus shown repeatedly. Proline residues are depicted in **bold**.

| **Gene** | **Patterns of positions of hydrophobic amino acids** | | | | |
| --- | --- | --- | --- | --- | --- |
|  | 1-4-7 | 1-4-1 | 1-5-7 | 1-5-1 | 1-5-2 |
| alpha2 | LRDILGF | LRDILGFL  ILDINKKL  LFSICCNL | LRDILGF | LRDILGFL  INKKLFSI  ICCNL**P**KL  FKSSILDI | IKDLLNPQI  ITDEFKSSI |
| Cti6 | VEKVDTI  LSMMDDL | VEKVDTIY  MDDLTREL  YNGYNESL | YNESLSM  LTRELLL | YNESLSMM  LTRELLLW |  |
| Dal80 | LTRL**P**NL  L**P**NLSTL | L**P**NLSTLL | YTSNIEL |  |  |
| Fkh1 | VNAYAKI | VQKLEVTI  WTYYVQKL | VKKNIDI | WTYYVQKL  IAREVNAY | VKKNIDIDL |
| Gal80 |  | IDMIVIAI  LKYLFVEW  YLFVEWAL | IVIAIQV | LKYLFVEW  IDMIVIAI | VEWALACSL |
| Mig1 | VQELETL | IRSL**P**L**P**F |  |  |  |
| Mot3 |  |  | ITDQFQF |  | ITDQFQFQL |
| Opi1 | LNILDRV  ISNVVTF  LKSIGRL  VTFYDEI | LDRVSNKI  VVTFYDEI  LKSIGRLL | ISNVVTF | ISNVVTFY  VVTFYDEI | VSNKIISNV |
| Rdr1 | LRLLNHI  VLQIIDI | LRLLNHIL  LVKLDQAW  WNVLSTSF | LYRRLRL  LDKNVVL  VLQIIDI  LSAAYAL  LSTSFQY | LLQALAAV  LYRRLRLL  LDQAWWNV  VVLQIIDI  LSAAYALV  LSTSFQYV | ICLSLYRRL  LNHILDKNV  LDQAWWNVL |
| Rfx1 | FLSVHYF  VHYFEFY | VHYFEFYL  YFIFS**P**SF | I**P**AKYFI | I**P**AKYFIF | FTRKI**P**AKY |
| Rox1 |  | L**P**QLSSQL | FNNNIVL | FNNNIVLM  LNNDLKRL  VSRSLSGL |  |
| Sko1 | LSSLLNL |  | LSSLLNL | LT**P**GLSSL | LT**P**GLSSLL |
| Ume6 | ISNVNDM |  | VLR**P**ILL |  | VLR**P**ILLRI |
| Ure2 | FSDMSHV | VSNLSNAL |  | LSNALRQV | INFEFSTGV |
| Xbp1 |  | FLKYASEV |  |  |  |
| Yox1 |  |  |  |  | LASRVKADI |
